# Physician Consultations, Prostate Cancer Knowledge, and PSA Screening of African American Men in the Era of Shared Decision-Making

**DOI:** 10.1177/1557988318763673

**Published:** 2018-04-16

**Authors:** Leanne Woods-Burnham, Laura Stiel, Colwick Wilson, Susanne Montgomery, Alfonso M. Durán, Herbert R. Ruckle, Rupert A. Thompson, Marino De León, Carlos A. Casiano

**Affiliations:** 1 Center for Health Disparities and Molecular Medicine, Department of Basic Sciences, Loma Linda University School of Medicine, Loma Linda, CA, USA; 2 Loma Linda University School of Behavioral Health, Loma Linda, CA, USA; 3 Oakwood University, Huntsville, AL, USA; 4 University of Michigan School of Nursing, Ann Arbor, MI, USA; 5 Department of Surgical Urology, Loma Linda University School of Medicine, Loma Linda, CA, USA; 6 Department of Surgical Urology, Wyckoff Heights Medical Center, Brooklyn, NY, USA; 7 Department of Medicine, Loma Linda University School of Medicine, Loma Linda, CA, USA

**Keywords:** African American, prostate cancer health disparities, prostate-specific antigen, shared decision-making, health knowledge

## Abstract

African American (AA)/Black men are more likely to develop aggressive prostate cancer (PCa), yet less likely to be screened despite guidelines espousing shared decision-making regarding PCa screening and prostate-specific antigen (PSA) testing. Given the documented racial disparities in PCa incidence and mortality, engaging interactions with physicians are especially important for AA/Black men. Thus, this study evaluated occurrence of physician–patient conversations among AA/Black men, and whether such conversations were associated with PCa knowledge. We also quantified the serum PSA values of participants who had, and had not, discussed testing with their physicians. Self-identified AA/Black men living in California and New York, ages 21–85, donated blood and completed a comprehensive sociodemographic and health survey (*n* = 414). Less than half (45.2%) of participants had discussed PCa screening with their physicians. Multivariate analyses were used to assess whether physician–patient conversations predicted PCa knowledge after adjusting for key sociodemographic/economic and health-care variables. Increased PCa knowledge was correlated with younger age, higher income and education, and having discussed the pros and cons of PCa testing with a physician. Serum PSA values were measured by ELISA. Higher-than-normal PSA values were found in 38.5% of men who had discussed PCa screening with a physician and 29.1% who had not discussed PCa screening. Our results suggest that physician–AA/Black patient conversations regarding PCa risk need improvement. Encouraging more effective communication between physicians and AA/Black men concerning PCa screening and PSA testing has the potential to reduce PCa health disparities.

Prostate cancer (PCa) is the most commonly diagnosed male cancer in the United States (Siegel, Miller, & Jemal, 2017), and African American (AA) men are 2.5 times more likely to die from this malignancy than European American (EA) men (Chornokur, Dalton, Borysova, & Kumar, 2011; Siegel et al., 2017). Early detection through timely screening and optimal treatment options improve overall survival, yet AA and other Black men of African ancestry are not as likely to receive these health advantages as EA men (Benjamins et al., 2016; Chornokur et al., 2011; DeSantis et al., 2016; Kelly et al., 2017; Kinlock et al., 2016; Mahal et al., 2017; Siegel et al., 2017; 2011; Singh & Jemal, 2017). With the implementation of the Affordable Care Act, the gap in access to high-quality health care, timely diagnosis, and optimal treatment has narrowed between AAs and EAs (Bach et al., 2002; Barnett & Vornovitsky, 2016; DeNavas-Walt et al., 2013). However, even under equal access to health care, disparities in PCa treatment and screening options still persist (Bach et al., 2002; Barnett & Vornovitsky, 2016; DeNavas-Walt et al., 2013; Ward et al., 2004; Ward et al., 2008).

PCa diagnosis involves prostate-specific antigen (PSA) screening (American Academy of Family Physicians, 2017; American Cancer Society, 2017; Halbert et al., 2017; Han et al., 2013; Moyer, 2012; Powell, Vigneau, Bock, Ruterbusch, & Helibrun, 2014; Saltzman et al., 2015; Shen & Kumar, 2016; Siegel et al., 2017). Circulating serum PSA levels are considered abnormal when detected above 4 ng/ml (American Academy of Family Physicians, 2017; Moyer, 2012). While the U.S. Food and Drug Administration approved the use of PSA testing in conjunction with digital rectal examination (DRE) to screen asymptomatic men for PCa in 1994, the U.S. Preventive Services Task Force (USPSTF) issued a recommendation against PSA-based screening in 2012 (Moyer, 2012). This recommendation was based on the assumption that, for most men, screening has no net benefit or the harms may outweigh the benefits (Shen & Kumar, 2016; Moyer, 2012). The recommendation influenced the current American Academy of Family Physicians’ overarching stance to “not routinely screen for PCa using a PSA test or DRE” (American Academy of Family Physicians, 2017). However, decreased screening differentially affects “patient populations under consideration” which includes AA men (Moyer, 2012). This is because the USPSTF report acknowledged that no firm conclusions about the benefits-to-harm ratio of PSA screening can be drawn in AA men due to their limited representation in the clinical trials that supported the recommendation against PSA screening (Moyer, 2012). A more recent study that used Surveillance, Epidemiology, and End Results (SEER) data to investigate survival disparities between AA (*n* = 23,782) and EA (*n* = 188,937) men comparing pre-PSA testing era to current-PSA testing era provided a compelling case for continued aggressive PSA testing for AA men (Powell et al., 2014). Additionally, frequent and early PSA testing has been suggested for AA men in order to reduce racial disparities in PCa mortality (Powell et al., 2014; Saltzman et al., 2015).

Previous USPSTF guidelines recognized that before offering PSA screening, shared decision-making should occur through an engaged physician–patient conversation that enables informed choice based on patient preferences (Moyer, 2012). The American Academy of Family Physicians currently advises that physicians offering PSA screening be “prepared to engage in shared decision-making that enables an informed choice by patients” (American Academy of Family Physicians, 2017). The American Cancer Society (ACS) also encourages informed decision-making and recommends PCa screening at age 50 for men at average-risk, 45 for men at high-risk, and 40 for men at higher-risk (American Cancer Society, 2017). ACS includes AA men in the high-risk category and recommends repeated annual PCa screening for men with PSA levels >2.5 ng/ml (American Cancer Society, 2017). While informed decision-making is the current recommendation for PCa screening, recent studies highlight that AA men may not be making informed decisions about PCa screening (Davis et al., 2010; Feng et al., 2013; Halbert et al., 2017; Han et al., 2013; Hoffman et al., 2009; Leyva et al., 2016; McCormack et al., 2009). This is largely due to patients having limited knowledge of PCa screening and providers either not offering sufficient up-to-date information or not asking patients about their preferences (Davis et al., 2010; Hoffman et al., 2009; Leyva et al., 2016; McCormack et al., 2009). Therefore, there is a need for more engaging interactions regarding PCa screening between physicians and patients, especially for AA and other Black men of African ancestry, who are more likely to develop aggressive end-stage PCa at an earlier age (Powell, Bock, Ruterbusch, & Sakr, 2010). Knowledge of PCa and screening among AA/Black men may therefore play a critical role in reducing PCa health disparities.

AA/Black men in the United States comprise a heterogeneous population that includes both native- and foreign-born individuals, and nativity can affect individual health outcomes (Erving, 2011; Williams & Sternthal, 2010). Our survey data from a cohort of self-reported AA/Black men in two U.S. geographical regions focused on assessing men’s knowledge of PCa in light of clinical provider interactions. We explored factors potentially influencing PCa knowledge among AA/Black men, including whether their physicians had discussed PCa screening with them. Additionally, PSA values of participants were assessed to demonstrate the real-life value of PSA screening in this high-risk population.

## Methods

### Participant Cohort

Cross-sectional data were collected via Project C.H.A.N.G.E (Changing Health for Adult Men with New and Great Experiences), in Riverside, CA in 2013 and Brooklyn, NY in 2014. Recruited through community outreach, a convenience sample of adult men either donated blood or completed a 141-item health survey, or both, after written informed consent. While all study participants self-identified as Black, some participants further self-identified as AA and others as Caribbean Black or African. For discussion purposes, we grouped them under the general term of AA/Black. This study was conducted under approval of Loma Linda University Institutional Review Board (OSR#5110343).

### Serum Collection

Blood was drawn by licensed staff and collected in red top vials. Collected blood rested at room temperature for 30 min to allow clotting. Serum was separated from blood cells by centrifugation, transferred to polypropylene tubes, and transported in dry ice for permanent storage at −80^ο^C.

### PSA ELISA

Human PSA ELISA Kits were purchased from Abnova (Taoyuan City, 320 Taiwan, catalog #KA0208). The 96-well ELISA plates were pre-coated with goat anti-PSA antibody for serum PSA detection. Following completion of the health fairs, sera from study participants who donated blood samples were added to the wells and circulating PSA was allowed to bind to the immobilized antibody. Wells were washed to remove unbound PSA. Monoclonal anti-PSA-horseradish peroxidase conjugate was then added to each well and allowed to bind PSA. Wells were washed and TMB (3,3’,5,5’ tetramethylbenzidine) reagent (provided in kit) was added to each well followed by incubation. Color development was interrupted with Stop Solution (provided in kit), and absorbance was measured by spectrophotometer at 450 nm, with PSA concentration directly proportional to color intensity. PSA values were calculated from a standard curve generated using PSA standards provided with the kit. PSA measurements were performed in duplicates for all serum samples. To ensure IRB compliance, individual PSA values were de-identified and not disclosed to study participants.

### Statistical Analysis

Sociodemographic, socioeconomic, and health-care variables were evaluated using validated items from national surveys (LaVeist et al., 2009; Deibert et al., 2007). Age was assessed as a continuous variable ranging from 21 to 85 years. Income was originally a categorical variable with 23 groups. We created a continuous distribution of income by constructing a new variable in which we estimated the midpoint of each group (Treiman, 2009). We estimated the lowest group (0 to $5000) at $1000 and the top income group (more than $350,000) at $750,000. The resulting approximate income distribution was skewed. To minimize the skew, we took the log of the distribution and used this log transformation in our analyses. Education was coded as a categorical variable with three levels: high school graduate or below; some college or associate’s degree; and college graduate and above. To measure participant trust of health-care providers and organizations, an 18-item adapted version of the Medical Mistrust Index was used (LaVeist et al., 2009). Items were summed and normalized to the original 4-point Likert scale, with higher scores indicating higher mistrust. Knowledge of PCa was assessed using 11 items modified from Deibert et al.’s scale (Deibert et al., 2007). For each true/false question, a correct response was coded with a value of “1,” while an incorrect response or unanswered item was coded with a “0.” All items were then summed. Thus, a higher score represents higher PCa knowledge. Respondents who did not complete any knowledge questions or other relevant, nearby survey sections were excluded from the analysis (*n* = 3). Categorical variables with yes/no responses included whether participants had health insurance, were told by a physician that they had PCa, or ever discussed the pros and cons of PCa screening with a physician. Descriptive analysis was performed to explore distributions and describe the sample (Table 1). Multivariate analysis was conducted with the following variables: age, education (college graduate and above as the reference group), log income, health insurance, medical mistrust, ethnicity, whether a physician had told the respondent that he had PCa, and whether a physician had discussed with the respondent the pros and cons of testing. Hierarchical models were developed, but only the final model is presented (Table 2). Prior to analysis, data were screened for linearity, normality, and homoscedasticity. Except for income, no transformations were made. Based on Mahalanobis testing, no outliers were excluded. Statistical analysis was performed using IBM SPSS 23 (IBM SPSS Statistics for Macintosh, 2013).

**Table 1.** Descriptive Statistics^a^.

Variables	Range	Mean or percentage	Standard deviation
**Dependent variable**			
Prostate cancer knowledge	0–11	7.9	2.2
**Demographic variables**			
Foreign-born Blacks	0–100	60.6	
Age	21–85	48.9	14.5
**Socioeconomic status**			
High school graduate or below	0–100	29.0	
Some college or associate’s degree	0–100	38.6	
College graduate and above	0–100	32.4	
Logged income	6.91–13.53	10.6	1.2
Has health insurance	0–100	68.1	
**Medical beliefs and experience**			
Medical Mistrust Scale	1–4	2.5	0.38
Diagnosed with prostate cancer	0–100	3.9	
Doctor discussed screening pros/cons	0–100	45.2	
Tested for prostate cancer	0–100	39.1	
Had PSA test	0–100	21.0	
Had digital rectal exam	0–100	34.3	

*Note.* ^a^*n* = 414. PSA = prostate-specific antigen.

**Table 2.** Multivariate Modeling of Predictors of Prostate Cancer Knowledge (*n* = 363).

Variable	Coefficient	Standard error	95% CI		*p* value	Referent
Ethnicity	.069	.2128084	−.3492875	.487776	0.745	U.S.-born Blacks
Age	−.024	.0081818	−.0403466	−.0081642	0.003	Not applicable
Education						
High school graduate or below	−.983	.2826495	−1.538938	−.4271609	0.001	College graduate and above
Some college or associate’s degree	−.730	.2417114	−1.20496	−.254209	0.003	College graduate and above
Income	.373	.0906024	.1945511	.5509279	0.000	Not applicable
Health insurance	−.007	.2307852	−.4609131	.4468605	0.976	No health insurance
Medical mistrust	.306	.2631934	−.2114296	.8238191	0.245	Not applicable
Told has prostate cancer	−.535	.5159006	−1.549288	.4799626	0.301	Yes, told has prostate cancer
Discussed pros/cons of testing	.482	.2312566	.0272432	.9368714	0.038	Discussed pros/cons
Constant	5.315	1.306724	2.745492	7.885378	0.000	
*R* ^2^	0.16					

## Results

### Univariate Analyses

Only participants who provided written consent, donated blood, and completed the survey were included in our analyses (*n* = 414). Men with a previous prostate cancer diagnosis (16/414, 3.9%) were included in analyses. We evaluated ethnicity in two groups: U.S.-born (163/414) AA men and foreign-born (251/414) Black men living in the United States. Within the foreign-born group, 85.8% (215/251) of participants were from the Caribbean West Indies. Descriptive characteristics of study participants are shown in Table 1. Within the cohort, 45.2% (187/414) of participants reported having spoken with their physicians about the pros and cons of PCa screening.

### Multivariate Analyses

Regression analysis (Table 2) assessed the relative contribution of correlates on PCa knowledge. Results indicate that PCa knowledge was inversely associated with age and positively associated with income. Compared to those with a high school degree or less, men who had a college degree or above, or had some college education or associate’s degree, reported higher PCa knowledge scores. Importantly, after adjusting for the variables identified in Table 2, men who discussed the pros and cons of testing with their physicians reported higher PCa knowledge. Nonsignificant variables included not having health insurance, level of medical mistrust, having been told he has PCa, ethnicity, and the length of stay in the United States of the foreign-born participants (data not shown).

### PSA Values in the Context of PCa Screening Conversations With Physician

**Figure 1.**
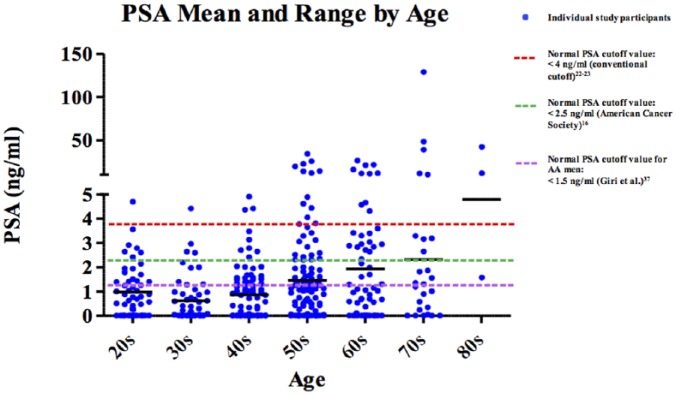
PSA values of AA/Black study participants differentiated by reported normal cutoffs. Serum PSA levels in sera of the study participants were determined by ELISA. As expected, average PSA levels increased with age, with PSA of men in their 30s averaging 0.8 ng/ml and PSA of men in their 80s averaging 16.4 ng/ml. We identified participants who had higher-than-normal PSA in the context of differing numerical values for what is considered higher-than-normal PSA levels for AA men. While the conventional cutoff for higher-than-normal PSA levels is 4 ng/ml, the American Cancer Society currently advises repeat screening for men with PSA levels greater than 2.5 ng/ml, and one study suggests 1.5 ng/ml as a predictor for PCa in AA men (American Cancer Society, 2017; Giri et al., 2009). Our results revealed that 33.3% of study participants had higher-than-normal PSA levels. Of these, 12.1% of participants (50/414) had PSA levels >4 ng/ml, 9.4% (39/414) had detectable PSA levels between 2.5–3.9 ng/ml, and 11.8% (49/414) had detectable PSA levels between 1.5–2.49 ng/ml. PSA = prostate-specific antigen; AA = African American; enzyme-linked immunosorbent assay; PCa = prostate cancer.

**Figure 2.**
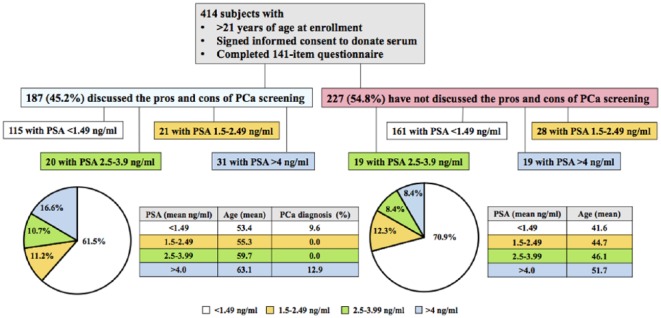
PSA values of study participants who had discussed PCa screening with their physicians vs. those who had not. Diagram illustrating the percentage of participants with PSA values considered as high-risk separated into groups by those who had discussed the pros and cons of PCa screening versus those who had not. Of the total study participants, 54.8% (227/414) had never discussed the pros and cons of PSA testing with their physicians. Of these, 29.1% (66/227) had higher-than-normal PSA values as determined using the three cutoff values defined in Figure 1. Conversely, 45.2% (187/414) of study participants had discussed the pros and cons of PSA testing with their physicians. Of these, 38.5% (72/187) had higher-than normal PSA values as determined using the three cutoff values defined in Figure 1. PSA = prostate-specific antigen; PCa = prostate cancer.

ELISA was used to quantify serum PSA levels in men participating in the study. Average PSA levels increased with age, with levels in men in their 30s averaging 0.8 ng/ml and men in their 80s averaging 16.4 ng/ml (Figure 1). Results revealed that 12.1% (50/414) of participants had higher-than-normal PSA levels when using the conventional cutoff >4 ng/ml. Of these, 38.0% (19/50) had never discussed the pros and cons of PCa screening with a physician (Figure 2). Additionally, 9.4% (39/414) of all the men in the study cohort had detectable PSA levels between 2.5 and 3.9 ng/ml, and 48.7% (19/39) of these had not discussed the pros and cons of PCa screening with a physician (Figure 2). Further, 11.8% (49/414) of total participants had detectable PSA levels between 1.5 and 2.49 ng/ml, and of these, 57.1% (28/49) had never discussed the pros and cons of PCa screening with a physician (Figure 1). Thus, 33% (138/414) of all participants had PSA values above 1.5 ng/ml, and of these, 47.8% (66/138) had not discussed PCa screening with their physicians. The remaining 66.7% (276/414) had PSA levels <1.5 ng/ml (Figure 2).

## Discussion

In light of the disproportionately high disparities in PCa incidence and mortality affecting AA/Black men, recent recommendations espouse earlier screening for this group (American Cancer Society, 2017; Powell et al., 2014; Saltzman et al., 2015). USPSTF guidelines are also currently undergoing a process of revision (U.S. Preventive Services Task Force, 2017), although a full recommendation statement has not been finalized. The current draft under consideration includes a level C recommendation that clinicians inform men ages 55–69 about the potential benefits and harms of PSA-based screening for PCa, with a recommendation against PSA-based screening in men 70 years and older. A widespread endorsement of clinician conversations regarding PCa screening would be beneficial as studies have shown that a better understanding of PCa by AA/Black men is critical for reducing these disparities since knowledge of this disease strongly influences informed decision-making (Leyva et al., 2016; McCormack et al., 2009; Ross, Powe, Taylor, & Howards, 2008).

Several factors are associated with PCa knowledge among AA/Black men, including physician consultation (Ross et al., 2008; Ross et al., 2011). An important component of physician conversations with patients regarding PCa screening involves discussing the potential benefits and harms of testing (Carpenter et al., 2010; Halbert et al., 2017; Leyva et al., 2016; Ross et al., 2008; Ross et al., 2011; Ward et al., 2004). Ideally, these conversations should culminate in increased PCa patient knowledge to help steer choices regarding screening and treatment (Davis et al., 2010; Feng et al., 2013; Hoffman et al., 2009; Leyva et al., 2016; McCormack et al., 2009). However, AA/Black men are less likely to receive sufficient information from their physicians about PSA testing to make an informed decision (Davis et al., 2010; Hoffman et al., 2009; Leyva et al., 2016).

Recognizing these concerns, this study evaluated the occurrence of physician–patient conversations within an AA/Black men cohort and assessed whether this translated into an increase in PCa knowledge and PSA testing. While Han et al. (Han et al., 2013) found that physician–patient conversations about the advantages and disadvantages of PSA testing were positively associated with Black race or Hispanic ethnicity, our study represents a novel step in that it focuses specifically on AA/Black men. Additionally, it assesses whether having discussed PCa screening with a physician is associated with higher PCa knowledge. Our study approach was also unique in that the responses were aligned with newly quantified PSA levels. We identified participants who had higher-than-normal PSA, recognizing that PCa experts assign differing cutoff values for what is considered higher-than-normal PSA levels for AA/Black men. For instance, while the conventional cutoff for higher-than-normal PSA is 4 ng/ml, ACS now recognizes PSA >2.5 ng/ml as reason for repeat annual screening, and one study suggests >1.5 ng/ml for AA men (American Cancer Society, 2017; Moyer, 2012; American Academy of Family Physicians, 2017; Giri et al., 2009). Therefore, we determined the percentage of men under these PSA cutoff values separately. This study is also the first to report high-risk AA/Black men with higher-than-normal PSA values who had yet to discuss the pros and cons of PCa testing with their physicians. While elevated PSA does not inevitably predict PCa, these findings were distinctive as it has been reported that 1 in 4 AA/Black men will be diagnosed with PCa, yet 1 in 3 of our AA/Black participants had elevated PSA levels, which could be indicative of underlying PCa (Lloyd et al., 2015). We cannot rule out, however, that elevated PSA levels in some participants may be unrelated to PCa.

Multivariate analysis revealed that PCa knowledge increased as age decreased, as income and education increased, and in men who had discussed the pros and cons of testing with their physicians. This suggests that increased physician interaction with less-educated and lower-income men is critical, given that these groups are less likely to access health care or navigate their discussions with physicians as easily as their peers with higher income and education. Our findings also suggest that AA/Black men in their 40s may not have the knowledge they need to consider their high risk for PCa while making a decision about screening.

As expected, PSA values in our AA/Black male cohort increased with age. Approximately one-third of participants had PSA values that could be considered higher-than-normal; however, over half of the men had never discussed the pros and cons of PSA testing with their physicians. This is problematic as this high-risk population should be well-informed about PCa risk and screening options. Our results reveal that the physician–patient conversations may not be occurring frequently enough in a population with existing higher-than-normal PSA values, which includes AA/Black men under 40 years old. However, we recognize that there are no current recommendations for PCa screening for men in their 20s and 30s.

A limitation of this study is that participants were not instructed to accurately identify the type of physician or health care provided they interacted with (e.g., family physician, urologist, nurse practitioner), which may have impacted the emphasis placed on a PCa screening conversation. Because of this limitation, we did not explore measures of physician competency that may have impacted the efficacy of these conversations. Another potential limitation is that the study was conducted within a church-based (Seventh-Day Adventist) population and urban areas of California (Riverside) and New York (Brooklyn), which are likely to attract men who are more aware about their health and about cancer prevention (Beeson et al., 1989; Heuch et al., 2005; Jacobsen, Knutsen, & Fraser, 1998). To counterbalance the potential confounding factor of religion on survey responses as well as increase community involvement, we also recruited non-church affiliated men through local Black-owned barbershops for our Riverside event and from community organizations in Brooklyn. Nevertheless, consistent with previous findings, faith-based organizations are promising venues for health promotion in AA/Black communities (Maynard, 2017; Sattin et al., 2016; Woods et al., 2013). We also experienced during the course of our study that these organizations provide an excellent venue and mechanism for the recruitment of AA/Black men for community-based participatory research.

Encouragingly, the physician–patient conversations that are occurring regarding PCa screening appear to be effective, as verified by subsequent PCa knowledge assessment. There is room for improvement, however, as we found that for many men who exhibited high PSA values and had discussed with their physicians the pros and cons of PCa screening, these discussions did not translate to actual PSA testing in 24% of this subgroup of men. This study further highlights the continued need for effective communication between physician and patient regarding prostate health and PCa screening and for better provider education about the special needs of AA men, which may not have been adequately addressed under existing procedural recommendations.
